# Identification of miR‐150‐5p in Human Amniotic Membrane Mesenchymal Cell‐Derived Extracellular Vesicles as a Novel Mechanism Driving Cardioprotection

**DOI:** 10.1111/eci.70212

**Published:** 2026-04-28

**Authors:** Nunzio Alcharani, Laura Tesoro, Javier Díez‐Mata, José Luis Zamorano, Marta Saura, Maite Iglesias, Carlos Zaragoza

**Affiliations:** ^1^ Unidad Mixta de Investigación Cardiovascular Universidad Francisco de Vitoria, Hospital Ramon y Cajal (IRYCIS) Madrid Spain; ^2^ Departamento de Cardiología Hospital Universitario Ramón y Cajal (IRYCIS) Madrid Spain; ^3^ Centro de Investigación Biomédica en Red de Enfermedades Cardiovasculares (CIBERCV) Instituto de Salud Carlos III (ISCIII) Madrid Spain; ^4^ Unidad de Fisiología, Departamento de Biologia de Sistemas Universidad de Alcalá (IRYCIS), Alcala de Henares Madrid Spain; ^5^ Facultad de Ciencias Experimentales Universidad Francisco de Vitoria Madrid Spain

**Keywords:** acute myocardial infarction, cardioprotection, extracellular vesicles, HOXA4, human amniotic mesenchymal stem cells, ischemia/reperfusion, MIAT, miR‐150‐5p

## Abstract

**Background:**

Human amniotic membrane mesenchymal stem cells (hAMSCs) hold strong cardioprotective potential, yet their mechanisms of action remain largely elusive.

**Methods and Results:**

C57BL/6 mice were subjected to cardiac ischemia/reperfusion (I/R) and received intravenous (IV) 2 × 10^5^ hAMSCs at 2, 7 or 14 days post‐reperfusion. Cardiac function and MIAT/miR‐150/HOXA4 signalling were assessed. Mice treated 2 days post‐I/R markedly improved LVEF and reduced myocardial necrosis and fibrosis by Day 21. Minimal hAMSC engraftment, evidenced by SSEA‐4 immunostaining, suggests paracrine rather than direct cellular effects. Guided by GWAS implicating miRNAs in myocardial infarction, we identified miR‐150 as a key effector, finding that hAMSC upregulated cardiac miR‐150 and suppressed HOXA4, a profibrotic target in ischemic myocardium. In parallel, hAMSC treatment reduced cardiac MIAT, a lncRNA that sequesters miR‐150, uncovering a mechanism of cardioprotection via MIAT downregulation post‐reperfusion. Notably, CRISPR‐Cas9 miR‐150‐silenced hAMSC exhibited severely impaired cardioprotective effects compared to wild‐type cells, confirming the functional role of miR‐150. miR‐150 was identified as a key extracellular vesicle (EV) cargo released by hAMSC under hypoxic conditions, both in vitro and in hAMSC‐injected I/R mice. Strickingly, administration of miR‐150‐enriched EVs to mice recapitulated the therapeutic benefits of hAMSC, underscoring miR‐150‐5p as central mediator of hAMSC‐iduced cardioprotection.

**Conclusions:**

hAMSCs promote cardioprotection following I/R via the MIAT/miR‐150‐5p/HOXA4 axis, in which miR‐150‐5p plays a central role. These findings provide loss‐of‐function evidence about the therapeutic potential of hAMSC‐derived EVs as a novel cell‐free exosome‐based strategy for the treatment of acute myocardial infarction.

## Graphical abstract

Human amniotic membrane mesenchymal stem cells (hAMSCs) provide significant cardioprotection following myocardial ischemia/reperfusion injury. They improve cardiac function and reduce fibrosis by modulating the MIAT/miR‐150‐5p/HOXA4 signalling axis. Notably, CRISPR‐Cas9 silencing of miR‐150‐5p abolishes these benefits, while miR‐150‐5p‐enriched extracellular vesicles (EVs) successfully recapitulate the therapeutic effects. These findings highlight hAMSC‐derived EVs as a promising cell‐free, exosome‐based strategy for treating acute myocardial infarction through targeted miRNA delivery.

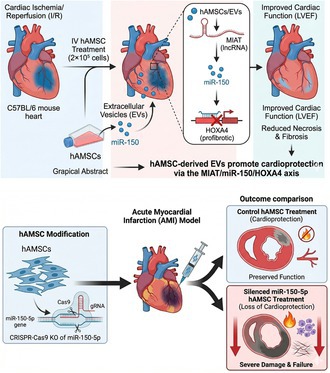

## 1 Introduction

Mesenchymal stem cells (MSCs) are multipotent stromal cells that can differentiate into osteogenic, chondrogenic, and adipogenic lineages [1, 2]. Beyond their differentiation potential, MSCs secrete a diverse array of bioactive factors that mediate immunomodulation, stimulate angiogenesis, and reduce fibrosis [3]. These properties render MSCs compelling candidates for tissue repair and regeneration, particularly in ischemic conditions such as acute myocardial infarction (AMI) [4, 5, 6]. Among the various sources of MSCs, those derived from human placenta exhibit unique advantages, including high proliferative capacity and immunomodulatory properties; however, they remain underexplored in clinical settings concerning myocardial infarction, which continues to impose a significant burden on a global scale, affecting both human resources and economic costs. Human amniotic membrane‐derived MSC (hAMSC) represent a promising and underexplored subset of MSCs with distinct biological advantages. hAMSC are distinguished by their high proliferative potential, immunomodulatory properties, and ability to thrive in hypoxic and inflammatory environments typical of ischemic myocardium [1, 2, 3, 7, 8]. Importantly, hAMSC release extracellular vesicles, such as exosomes, which are enriched with non‐coding RNAs, including microRNAs (miRNAs), lipids, and proteins that can influence the molecular pathways underlying myocardial injury and repair [9, 10, 11].

The long non‐coding RNA MIAT (Myocardial Infarction–Associated Transcript) has emerged as a pivotal regulator in the pathogenesis of cardiovascular disease. MIAT expression is significantly upregulated in infarcted myocardium [12, 13, 14], where it exacerbates myocardial injury by acting as a competing endogenous RNA (ceRNA) that sequesters miR‐150‐5p, a microRNA with established cardioprotective effects [15, 16]. By binding to miR‐150‐5p, MIAT attenuates the ability of miR‐150‐5p to repress its downstream target, HOXA‐4, a transcription factor implicated in the promotion of cardiomyocyte apoptosis and inflammatory responses [16]. This MIAT/miR‐150‐5p/HOXA‐4 regulatory axis has been identified as a critical contributor to post‐infarction myocardial remodelling and dysfunction. Accordingly, therapeutic strategies aimed at disrupting this axis, either by silencing MIAT or by augmenting miR‐150‐5p activity, hold promise for reducing myocardial apoptosis and improving cardiac function following ischemic injury.

On the other hand, exosomes derived from MSCs have gained recognition as key mediators of cell‐free regenerative therapies [17, 18]. These nanosized vesicles encapsulate and transport bioactive cargo to recipient cells, modulating signalling pathways critical for tissue repair. Among the miRNAs identified in MSC‐derived exosomes, miR‐150‐5p has emerged as a crucial regulator of cardiac fibrosis, inflammation, and angiogenesis [19, 20, 21]. As mentioned above, preclinical studies suggest that miR‐150‐5p exerts its cardioprotective effects by targeting key molecular pathways, including the MIAT/miR‐150‐5p/HOXA‐4 axis, which modulates extracellular matrix deposition and myocardial remodelling.

In this study, we investigate the therapeutic potential of hAMSC in a murine model of myocardial infarction, with a particular focus on their capacity to modulate the MIAT/miR‐150‐5p/HOXA‐4 regulatory axis. Building on emerging evidence that miR‐150‐5p plays a central role in cardioprotection by repressing pro‐apoptotic and pro‐fibrotic pathways, we hypothesize that hAMSC administration can enhance miR‐150‐5p expression in the injured myocardium, thereby disrupting the pathological interplay between MIAT and HOXA‐4.

## 2 Materials and Methods

### 2.1 Animal Model of Myocardial Ischemia/Reperfusion

All the surgical procedures were performed in the Experimental Surgery Department of the Hospital Universitario La Paz in Madrid (Spain). The procedures conformed to the Guide for the Care and Use of Laboratory Animals published by the US National Institutes of Health (NIH Publication No. 85‐23, revised 1985), the Animal Welfare Ethics Committee, the EU Directive on experimental animals (63/2010 EU), and the related Spanish legislation (RD 53/2013), PROEX 174.7/24.

Myocardial ischemia/reperfusion was induced as described [22]. In summary: mice were anesthetized with 3% sevoflurane and oxygen inhalation with a flow rate of 0.4 L/min until loss of righting reflex. Endotracheal intubation was performed using an intubation cannula for artificial ventilation (tidal volume: 260 μL/stroke, ventilation rate: 130 strokes per minute). The fourth left intercostal space was opened and widened using mouse chest retractors. The left ventricle was then exposed, and the LAD was occluded for 40 min by using a 6–0 silk suture and a 1 mm tube. Reperfusion was achieved through ligation release. After the procedure, the chest was closed, negative pressure was restored, and the skin was sutured.

### 2.2 Echocardiographic Assessment of Cardiac Function

Mice were anesthetized, and the Vivid Q ultrasound system from GE Healthcare (General Electric, Chicago, IL, USA), equipped with a 1.9–4 MHz scan head, was used to determine LV function. Parasternal long‐ and short‐axis‐view images of the heart were taken before the surgery, at the end of ischemia, and at the endpoint to determine LV function worsening and recovery. The parameters studied using the onsite software cardiac package were: systolic and diastolic interventricular septum thickness (IVS), systolic and diastolic left‐ventricle internal diameter (LVID), systolic and diastolic left‐ventricle posterior wall thickness (LVPW), left‐ventricle ejection fraction (LVEF), left ventricle shortening fraction (LVFS), heart rate (HR), and cardiac output (CO). Data acquisition and analysis were performed using the onsite cardiac software and VividQ ultrasound equipment, with the same operator analyzing all samples to minimize interobserver bias.

### 2.3 Cell Culture and Administration of hAMSC

hAMSC (Cellular Engineering Technologies, Coralville, USA, cat# HMSC‐AM‐100) were seeded on various culture surfaces at an initial concentration of 10^4^ cells/cm^2^. Cells were grown in DMEM (Dulbecco's Modified Eagle's Medium with 1 g/L glucose and without L‐glutamine) (Biowest, Nuaillé, France, cat# L0101) supplemented with 10% fetal bovine serum (FBS) (Biowest, Nuaillé, France, cat# S181B), Penicillin/Streptomycin/Fungizone (10 k/10 k/25 μg) (Lonza, Basel; Switzerland, cat# H317‐745E), 2 mM L‐glutamine (Sigma‐Aldrich, St. Louis, MO, USA, cat# G7513) and basic fetal growth factor (hFGF basic) (Gibco, cat# PHG0026). Cultures were maintained at 37°C in a 5% CO_2_ and 20% O_2_ atmosphere (normoxic conditions) or at 37°C in a 5% CO2 and 1% O2 (hypoxic conditions), with the medium replaced every 2–3 days. All cell handling procedures were previously reported [1], including cell morphology, proliferation kinetics (cumulative population doubling), immunophenotype (positive for CD105, CD90, CD73, CD44; negative for CD34, CD45), differentiation potential, and validation of hypoxia through HIF expression. Hypoxic conditions were established for 20–24 h (acute hypoxia), and their effectiveness was confirmed by analyzing hypoxia‐inducible factor (HIF) expression, as previously reported [1]. No morphological changes were observed under hypoxic conditions.

Cell morphology was routinely monitored by phase‐contrast microscopy, and no evidence of cellular detachment, structural alteration, or morphological deterioration was observed under normoxic or hypoxic conditions. Proliferative behaviour was evaluated based on confluency, as previously described [23]. Consistent with prior reports, no changes in cellular phenotype were detected.

After 90% confluence, cells were washed with PBS (Gibco, Grand Island, New York, United States, cat# 14190‐086), trypsinized (Sigma‐Aldrich, St. Louis, MO, USA, cat# T4174), and re‐seeded at the indicated density. We IV administered 2 × 10^5^ hAMSC cultured under normoxic conditions at the times indicated following MI, a dose consistent with previous murine studies employing similar cell numbers intravenously [23, 24] supporting both the feasibility and biological plausibility of this regimen. It is important to note that hAMSCs were maintained in serum‐free medium for 24 h to ensure consistency and to prevent contamination by serum‐derived components. Prior to injection, cells were washed three times with PBS and resuspended in PBS for tail‐vein delivery.

After I/R, at the times indicated, exosomes were harvested at 80%–90% confluency (10^6^ cells). NIH/3T3 (ATCC, Manassas, USA, cat# CRL‐1658) cells were grown in similar conditions to the hAMSC and used as a cellular control treatment. Animals treated with NIH/3T3 received the same number of cells.

EV uptake in the H9C2 cell line was performed under similar conditions. To block MSC‐EV release, MSCs were pre‐treated with 10 μM GW4869 (Cat# D1692, Sigma‐Aldrich, St. Louis, MO) for 24 h prior to secretome exposure and coculture with H9C2 [25].

### 2.4 Histological and Immunohistofluorescence Analysis

Hearts were embedded in paraffin, sectioned at 5 μm, deparaffinized, rehydrated, and stained using Haematoxylin/Eosin (HE) and Masson's Trichrome (MT) for subsequent analysis with the ImageJ software package, as described [22]. Additionally, confocal microscopy‐based immunohistochemistry analysis was performed on the same sections of infarcted hearts to assess the presence of hAMSC by a specific marker, SSEA4 (Stage‐specific embryonic antigen 4) (SSEA4 Monoclonal Antibody cat #: MC81370, Thermofisher, Pittsburgh, USA). Given that a mouse monoclonal antibody was used in murine heart sections, a ‘mouse‐on‐mouse’ staining protocol was employed to significantly reduce nonspecific background. In brief, heart sections were blocked with unconjugated goat anti‐mouse IgG Fab fragments diluted in PBS/BSA for 60 min, and then washed twice with PBS/0.05% Tween 20. Samples were incubated overnight with the corresponding primary antibody, washed three times, and then incubated with an HRP‐conjugated anti‐mouse secondary antibody. Sections were washed three times, and the signal was developed with the chromogen DAB.

hAMSC were transduced with pre‐packaged lentiviral particles encoding green fluorescent protein (GFP). For this purpose, lentiviral particles (donated by Dr. Ester Martín‐Villar, Universidad Francisco de Vitoria, Madrid, Spain) were used to transduce 70% confluent hAMSC grown in complete DMEM. Briefly, cells were incubated with viral supernatant supplemented with polybrene (4–8 μg/mL) for 24 h, and infected at multiplicity of infection (MOI) of 2 [26]. After 24 h, the cells were washed with PBS, replaced with fresh medium, and cultured for an additional 72 h. GFP expression was confirmed by fluorescence microscopy. GFP‐hAMSC were IV administered to mice at Day 2 post‐reperfusion. By Day 7 after I/R, the hearts were harvested and processed for histological analysis to assess GFP expression by fluorescence microscopy.

### 2.5 Nucleic Acid Isolation and Molecular Analyses

Blood and heart tissue samples underwent RNA extraction using the MIRNEASY kit (cat # 217084, Qiagen, Hilden, Germany) and the miRNeasy Serum/Plasma Advanced Kit (cat # 217204, Qiagen, Hilden, Germany) and the QIAzol Lysis reagent (cat # 79306, Qiagen, Hilden, Germany) for blood and tissue respectively. RNA integrity was verified using Agilent Tapestation. Quantitative real‐time PCR (qPCR) was employed to validate key miRNA targets using TaqMan assays (TaqMan Universal Master Mix II, no UNG, cat # 4440047, Thermofisher, Pittsburgh, USA), with relative expression calculated via the 2^−ΔΔCt^ method.

Maxima First Strand cDNA Synthesis Kit for RT‐qPCR (cat # K1641, Thermofisher, Pittsburgh, USA) was used for total cDNA synthesis, and TaqMan MicroRNA Reverse Transcription Kit (cat #: 4366597, Thermofisher, Pittsburgh, USA) was used to study mature miRNAs. Probes used were obtained from Thermofisher, Pittsburgh USA: miR‐150‐5p (hsa‐miR‐150, cat #: 4427975; ID: 000473), U6 (U6 snRNA, cat #: 4427975, ID: 001973), MIAT (Mm01196418_g1, cat #: 4351372), Hoxa4 (Mm01335255_g1, cat #: 4331182), GSK3β (Mm00444911_m1, cat #: 4331182) and ACTA2 (Hs00426835_g1, cat #: 4331182).

### 2.6 Exosome Isolation From Cell Culture and Plasma

Exosomes derived from hAMSC were isolated from 90% confluent cells, cultured in serum‐free medium for 24 h before processing. Exosomes were obtained from the conditioned medium of cultured cells by centrifugation at 3000 *g* for 10 min (Hitachi Centrifuge, Rotor R22A6) to remove cells. 10,000 *g* for 30 min (Hitachi Centrifuge, Rotor R22A6) to eliminate cellular debris and other extracellular vesicles. 100,000 *g* for 2 h (Beckman Ultracentrifuge, Rotor 70.1Ti) to isolate exosomes. After ultracentrifugation, the exosomes were resuspended in complete DMEM medium for culture applications, in PBS for animal treatment, in TRIzol for RNA analysis, and in RIPA buffer for protein analysis.

Exosome isolation from mouse plasma followed the same protocol, with an additional intermediate step involving filtration using a 0.22 μm Multipore Express filter. The validation of the isolated exosomes was performed using the Nanosight LM10 system and Immunoblotting (Malvern Panalytica) (Figure S1).

### 2.7 Gene Editing in hAMSC

The systematic study of genes associated with acute myocardial infarction was initially conducted through genome‐wide association studies (GWAS), using data from the NCBI GWAS Catalogue (https://www.ebi.ac.uk/gwas/search?query=myocardial%20infarction). Prioritized targets common across GWAS studies, focusing on MIAT, a gene regulated by miRNAs like miR‐150‐5p.

For gene editing of the target genes, the CRISPR/Cas9 system was employed using the px459 plasmid from GenScript (NJ, USA), which incorporates a puromycin resistance gene for selecting transformed clones. The CRISPR Design Tool platform was used to select two target sites with specificity in distinct regions of the locus corresponding to the miRNA‐150‐5p sequence (GGTTGGGAGACAGGGCCATG/CCCAACCCTTGTACCAGTGC). This strategy resulted in two similar plasmids, differing only in guide RNA sequence, improving miR‐150 locus deletion efficiency [27].

The plasmids obtained from GenScript Biotech were first amplified in DH5α competent cells (cat #: EC0112, Thermofisher, Pittsburgh, USA) and isolated using the HiSpeed Plasmid Midi Kit (cat #: 12643, Qiagen, Hilden, Germany). They were then transfected into hAMSC using the Lipofectamine RNAiMax system (cat. # 13778075, Thermo Fisher, Pittsburgh, USA). After 48 h, cells were selected with 1.5 μg/mL puromycin for 4–5 days. The selected cells were diluted and individually seeded in 96‐well plates (one cell/well) and grown for 2 weeks. Positive clones were collected, and gene silencing was confirmed using TaqMan qPCR with specific probes for miR‐150‐5p.

### 2.8 Immunoblotting

Exosome protein lysates were obtained using RIPA buffer (Thermo Fisher Scientific) supplemented with protease inhibitors (Roche, Mannheim, Germany). Protein concentration was quantified using the bicinchoninic acid assay (Thermo Fisher Scientific, Waltham, MA, United States). Equal amounts of protein (30 μg) were separated by sodium dodecyl sulfate–polyacrylamide gel electrophoresis (SDS‐PAGE) and transferred onto polyvinylidene difluoride (PVDF) membranes (Roche). Exosome characterization was performed using the Exosome Panel (Calnexin, CD9, CD63, CD81, Hsp70, TSG101) (Abcam, Cambridge, UK; cat# ab275018). Membranes were blocked with 3% bovine serum albumin (BSA) in Tris‐buffered saline containing 0.05% Tween‐20 (T‐TBS) for 1 h at room temperature and subsequently incubated overnight at 4°C with primary antibodies against CD9, CD63, CD81, TSG101, Hsp70 and Calnexin (1:500, Abcam). The following day, membranes were washed with T‐TBS and incubated for 1 h at room temperature with the corresponding horseradish peroxidase‐conjugated secondary antibodies (1:3000, Cell Signaling Technology). Proteins were identified by using ECL detection kit (Amersham, Buckinghamshire, United Kingdom; cat# RPN2209), with a ChemiDoc MP Imaging System (Bio‐Rad).

### 2.9 Statistical Analysis

All statistical analyses were performed using GraphPad Prism 9.0.1 (GraphPad Software Inc., La Jolla, California, USA) and SPSS 26 (IBM, Armonk, New York, USA). Data distribution was assessed using the D'Agostino–Pearson omnibus normality test and the Shapiro–Wilk test. All results are presented as mean ± standard deviation. For comparisons between two groups, either the Student's *t*‐test or the Mann–Whitney *U* test was applied. For multiple group comparisons, one‐way ANOVA or the Kruskal–Wallis test was performed, followed by Bonferroni's or Dunn's post hoc test, respectively. Statistical significance was considered at *p* < 0.05.

## 3 Results

### 3.1 hAMSC Induces Cardiac Protection in Mice Subjected to Myocardial Ischemia/Reperfusion

**FIGURE 1:**
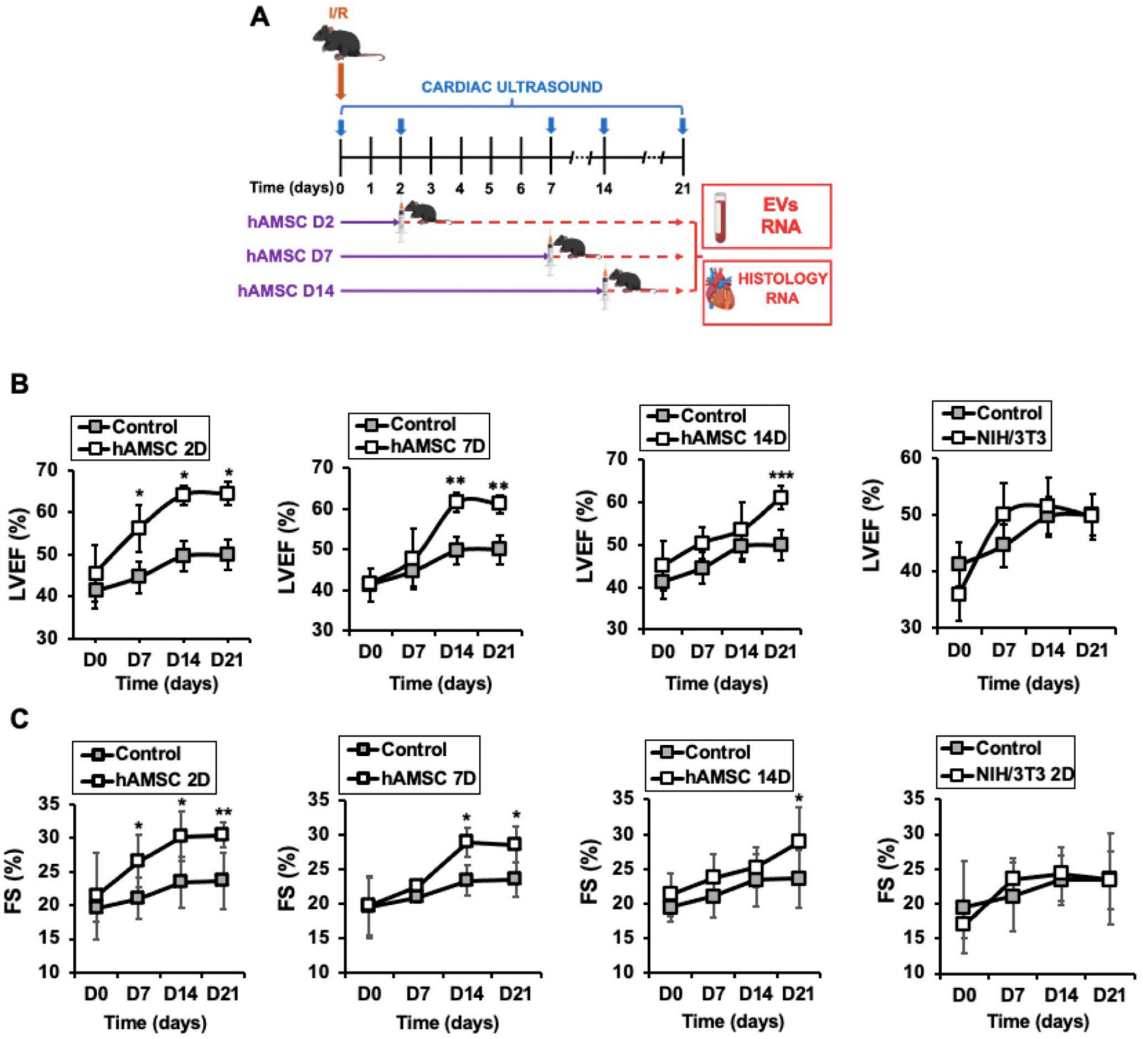
Human amniotic membrane–derived mesenchymal stromal cells (hAMSC) induce cardiac protection in mice subjected to myocardial ischemia/reperfusion (I/R). (A) Schematic representation of the study procedure. (B) Left ventricular ejection fraction (LVEF) at 0, 7, 14, and 21 days after I/R in mice treated with 2 × 10^5^ hAMSC after 2 (upper left), 7 (upper right), 14 (lower left) days post infarction, and in mice injected with NIH/3 T3 fibroblasts as a negative control. *N* = 10 mice/time point. Mean ± SD. Mice injected D2 post I/R *p < 0.001 D0 vs. D7, D14, D21. Mice injected D7 post I/R **p < 0.01 D0 vs. D14, D21. Mice injected D14 post I/R **p < 0.01 D0 vs. D21. C. Fractional shortening (FS) in the same mice. Mice injected D2 post I/R *p < 0.01 D0 vs. D7, D14, **p < 0.02 D0 vs. D21. Mice injected D7 post I/R *p < 0.01 D0 vs. D14, D21. Mice injected D14 post I/R *p < 0.01 D0 vs. D21.

The contribution of hAMSC in preventing adverse effects following myocardial IR was assessed by using three independent experimental groups (Figure 1A). Mice subjected to IR received a single IV injection of 2 × 10^5^ hAMSC at either 2 days (hAMSC D2), 7 days (hAMSC D7), or 14 days (hAMSC D14) post reperfusion. Control I/R animals received vehicle only. Mice injected with hAMSC exhibited a significant improvement in cardiac function at all indicated time points, as evidenced by a marked increase in left ventricular ejection fraction (Figure 1B) and fractional shortening (Figure 1C), when compared to Control I/R animals. The remaining echocardiographic parameters are depicted (Tables 1, 2, 3, 4). Interestingly, administration of control NIH/3 T3 cells (2 × 10^5^, as well) failed to confer any benefit, thereby reinforcing the specificity of the therapeutic effects mediated by hAMSC.

**TABLE 1:** Echocardiographic and hemodynamic evaluation of left ventricular structure and function across experimental groups following myocardial ischemia–reperfusion.

D0 post I/R							
Parameter	Control	NIH/3 T3	hAMSC 2D	hAMSC 7D	hAMSC 14D	hAMSC‐EVs 2D	Parameter
IVSD (mm)	0.748 ± 0.150	0.834 ± 0.109	0.770 ± 0.292	0.737 ± 0.074	0.800 ± 0.148	0.792 ± 0.138	IVSD (mm)
LVIDD (mm)	4.135 ± 0.642	3.957 ± 0.367	3.906 ± 0.742	4.090 ± 0.115	4.233 ± 0.355	3.944 ± 0.476	LVIDD (mm)
LVPWD (mm)	1.043 ± 0.381	1.030 ± 0.236	1.169 ± 0.393	0.888 ± 0.135	0.944 ± 0.206	1.152 ± 0.358	LVPWD (mm)
IVSS (mm)	0.970 ± 0.187	1.097 ± 0.159	0.934 ± 0.332	0.853 ± 0.135	1.040 ± 0.137	1.015 ± 0.171	IVSS (mm)
LVIDS (mm)	3.367 ± 0.776	2.900 ± 0.358	2.769 ± 0.999	3.146 ± 0.392	3.298 ± 0.366	3.211 ± 0.609	LVIDS (mm)
LVPWS (mm)	1.105 ± 0.531	1.270 ± 0.175	1.372 ± 0.588	1.064 ± 0.208	1.111 ± 0.205	1.221 ± 0.276	LVPWS (mm)
EF (%)	41.250 ± 3.955	36.000 ± 4.583	45.430 ± 6.800	41.750 ± 1.500	45.167 ± 5.845	41.75 ± 1.500	EF (%)
FS (%)	19.442 ± 1.959	16.967 ± 6.658	21.412 ± 6.468	19.678 ± 4.359	21.288 ± 3.061	20.337 ± 2.484	FS (%)
HR (bpm)	370.667 ± 54.375	425.333 ± 22.501	364.625 ± 110.916	386.000 ± 128.693	378.833 ± 70.884	392.114 ± 67.820	HR (bpm)
CO (mL/s)	53.328 ± 25.200	69.774 ± 20.672	66.140 ± 21.005	61.194 ± 16.567	74.269 ± 30.658	64.829 ± 19.744	CO (mL/s)

**TABLE 2:** Echocardiographic and hemodynamic evaluation of left ventricular structure and function across experimental groups at Day 7 following myocardial ischemia–reperfusion.

D7 post I/R							
Parameter	Control	NIH/3 T3	hAMSC 2D	hAMSC 7D	hAMSC 14D	hAMSC‐EVs 2D	Parameter
IVSD (mm)	0.797 ± 0.293	0.850 ± 0.219	0.819 ± 0.236	0.685 ± 0.174	0.850 ± 0.035	0.973 ± 0.429	IVSD (mm)
LVIDD (mm)	4.422 ± 0.984	4.193 ± 0.738	3.855 ± 1.253	4.436 ± 0.516	4.310 ± 0.315	3.913 ± 1.155	LVIDD (mm)
LVPWD (mm)	0.863 ± 0.249	0.990 ± 0.148	0.979 ± 0.257	0.857 ± 0.171	0.883 ± 0.189	1.007 ± 0.394	LVPWD (mm)
IVSS (mm)	1.235 ± 0.554	1.134 ± 0.070	1.171 ± 0.297	0.923 ± 0.070	1.065 ± 0.298	1.210 ± 0.096	IVSS (mm)
LVIDS (mm)	3.575 ± 0.431	3.956 ± 0.870	3.178 ± 0.387	3.800 ± 0.640	3.640 ± 0.568	3.383 ± 0.372	LVIDS (mm)
LVPWS (mm)	0.918 ± 0.356	1.087 ± 0.233	1.094 ± 0.366	0.942 ± 0.170	0.937 ± 0.179	1.033 ± 0.337	LVPWS (mm)
EF (%)	44.580 ± 3.730	50.0 ± 5.657	56.200 ± 5.650 ****	47.667 ± 7.371	50.400 ± 3.650	51.000 ± 6.083	EF (%)
FS (%)	21.011 ± 2.994	23.566 ± 4.919	26.488 ± 3.875 **	22.466 ± 0.816	23.754 ± 3.403	22.000 ± 2.646	FS (%)
HR (bpm)	396.750 ± 71.386	440.333 ± 18.475	382.375 ± 119.378	392.000 ± 58.988	450.250 ± 25.966	332.333 ± 56.722	HR (bpm)
CO (mL/s)	47.915 ± 26.699	44.847 ± 17.624	42.951 ± 13.389	61.090 ± 14.208	63.935 ± 26.918	46.877 ± 23.118	CO (mL/s)

*Note:* **p < 0.01 FS Control vs hAMSC 2D. ****p < 0.001 EF Control vs hAMSC 2D.

**TABLE 3:** Echocardiographic and hemodynamic evaluation of left ventricular structure and function across experimental groups at Day 14 following myocardial ischemia–reperfusion.

D14 post I/R							
Parameter	Control	NIH/3 T3	hAMSC 2D	hAMSC 7D	hAMSC 14D	hAMSC‐EVs 2D	Parameter
IVSD (mm)	0.915 ± 0.274	0.890 ± 0.062	0.913 ± 0.336	0.852 ± 0.121	0.867 ± 0.196	0.967 ± 0.351	IVSD (mm)
LVIDD (mm)	4.520 ± 0.536	4.230 ± 0.759	4.233 ± 0.555	4.590 ± 0.772	4.700 ± 0.709	4.137 ± 0.423	LVIDD (mm)
LVPWD (mm)	0.830 ± 0.400	0.892 ± 0.250	0.895 ± 0.254	0.798 ± 0.151	0.850 ± 0.092	0.867 ± 0.134	LVPWD (mm)
IVSS (mm)	1.253 ± 0.330	1.280 ± 0.248	1.328 ± 0.289	1.162 ± 0.195	1.190 ± 0.323	1.333 ± 0.438	IVSS (mm)
LVIDS (mm)	3.438 ± 0.578	3.217 ± 0.709	3.145 ± 0.716	3.257 ± 0.236	3.387 ± 0.387	3.070 ± 1.007	LVIDS (mm)
LVPWS (mm)	0.888 ± 0.240	0.970 ± 0.246	0.917 ± 0.312	0.843 ± 0.064	0.905 ± 0.304	0.927 ± 0.107	LVPWS (mm)
EF (%)	49.670 ± 3.500	51.500 ± 4.949	64.140 ± 2.270 ****	61.500 ± 2.380 ****	53.500 ± 6.661	57.333 ± 2.517 *	EF (%)
FS (%)	23.410 ± 3.794	24.273 ± 3.512	30.230 ± 3.739 **	28.986 ± 2.168 *	25.216 ± 2.876	25.667 ± 2.082	FS (%)
HR (bpm)	401.167 ± 95.315	444.333 ± 84.311	396.900 ± 64.518	452.200 ± 62.271	468.333 ± 100.580	349.667 ± 58.0718	HR (bpm)
CO (mL/s)	44.942 ± 25.056	40.560 ± 14.105	38.323 ± 24.724	48.250 ± 18.439	47.605 ± 7.146	41.897 ± 14.143	CO (mL/s)

*Note:* *p < 0.03 EF Control vs hAMSC‐EVs 2D; *p < 0.03 FS Control vs hAMSC 7D; **p < 0.01 FS Control vs hAMSC 2D; ****p < 0.001 EF Control vs hAMSC 2D; ****p < 0.001 EF Control vs hAMSC 7D.

**TABLE 4:** Echocardiographic and hemodynamic evaluation of left ventricular structure and function across experimental groups at Day 21 following myocardial ischemia–reperfusion.

D21 post I/R							
Parameter	Control	NIH/3 T3	hAMSC 2D	hAMSC 7D	hAMSC 14D	hAMSC‐EVs 2D	Parameter
IVSD (mm)	0.923 ± 0.206	0.900 ± 0.165	0.976 ± 0.202	0.857 ± 0.140	0.921 ± 0.161	0.793 ± 0.216	IVSD (mm)
LVIDD (mm)	4.638 ± 0.757	4.922 ± 0.788	4.538 ± 0.580	4.782 ± 0.367	4.669 ± 0.586	4.567 ± 0.576	LVIDD (mm)
LVPWD (mm)	0.703 ± 0.276	0.797 ± 0.133	0.720 ± 0.177	0.685 ± 0.192	0.822 ± 0.178	0.770 ± 0.110	LVPWD (mm)
IVSS (mm)	1.278 ± 0.447	1.320 ± 0.209	1.641 ± 0.347	1.264 ± 0.224	1.333 ± 0.388	1.353 ± 0.649	IVSS (mm)
LVIDS (mm)	3.367 ± 0.776	2.900 ± 0.358	2.769 ± 0.999	3.146 ± 0.392	3.298 ± 0.366	3.023 ± 0.260	LVIDS (mm)
LVPWS (mm)	0.783 ± 0.192	0.880 ± 0.252	0.724 ± 0.176	0.730 ± 0.082	0.825 ± 0.110	0.877 ± 0.215	LVPWS (mm)
EF (%)	50.000 ± 3.560	49.667 ± 4.041	64.500 ± 2.718 ****	60.750 ± 7.371 ****	61.200 ± 3.421 ****	58.667 ± 6.351 **	EF (%)
FS (%)	23.566 ± 4.147	23.409 ± 6.506	30.400 ± 1.838 ***	28.633 ± 2.582 *	28.845 ± 5.127*	26.667 ± 4.619	FS (%)
HR (bpm)	432.000 ± 69.120	502.800 ± 68.064	431.273 ± 44.439	469.750 ± 21.329	477.000 ± 31.943	394.667 ± 33.828	HR (bpm)
CO (mL/s)	31.499 ± 10.813	34.927 ± 5.372	27.276 ± 16.430	31.010 ± 6.774	33.267 ± 12.083	27.070 ± 14.142	CO (mL/sec)

*Note:* *p < 0.03 FS Control vs hAMSC 7D; *p < 0.03 FS Control vs hAMSC 14D; **p < 0.01 EF Control vs hAMSC‐EVs 2D; ***p < 0.01 FS Control vs hAMSC 2D; ****p < 0.001 EF Control vs hAMSC 2D; *p* < 0.001 EF Control vs hAMSC 7D; *p* < 0.001 EF Control vs hAMSC 14D.

Collectively, these findings underscore the therapeutic potential of hAMSC in reducing cardiac dysfunction and strengthen the rationale for their use as a targeted, time‐sensitive intervention for myocardial infarction.

**FIGURE 2:**
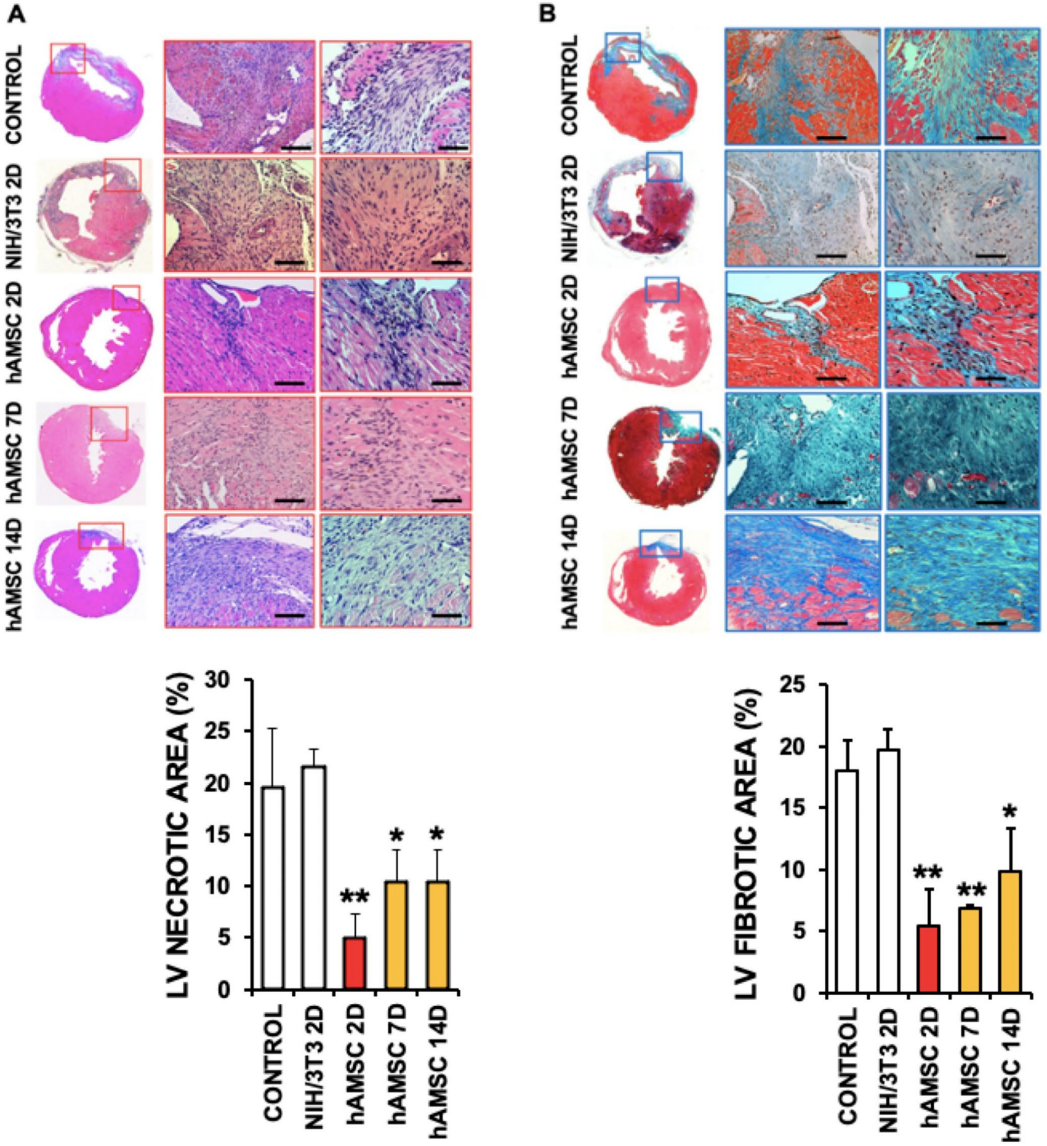
Human amniotic membrane–derived mesenchymal stromal cells (hAMSC) reduce cardiac necrosis and fibrosis in the hearts of mice 21 days after reperfusion. Bright field microscopy images of cardiac sections of hearts isolated from mice after 21 days of I/R, and injected with NIH/3 T3 fibroblast or hAMSC at the times indicated after reperfusion, and stained with Haematoxylin/Eosin (A), and Masson's Trichome (B). *N* = 5 mice/group. Mean ± SD. *p < 0.001 Control vs. hAMSC 2D, **p < 0.02 Control vs. hAMSC 7D, 14D. Scale bars = 100 μm.

Likewise, haematoxylin/eosin (H&E) staining of cardiac sections from the same mice revealed a marked reduction in inflammatory foci (Figure 2A), and consistently, Masson's Trichrome staining also demonstrated a notable decrease in cardiac fibrosis by Day 21 following hAMSC administration (Figure 2B). Of particular interest, mice that received early hAMSC treatment (Day 2 post‐IR) exhibited the most pronounced reduction in fibrotic tissue, highlighting the therapeutic advantage of timely intervention.

### 3.2 Cardioprotection by hAMSC Is Independent of Myocardial Engraftment

**FIGURE 3:**
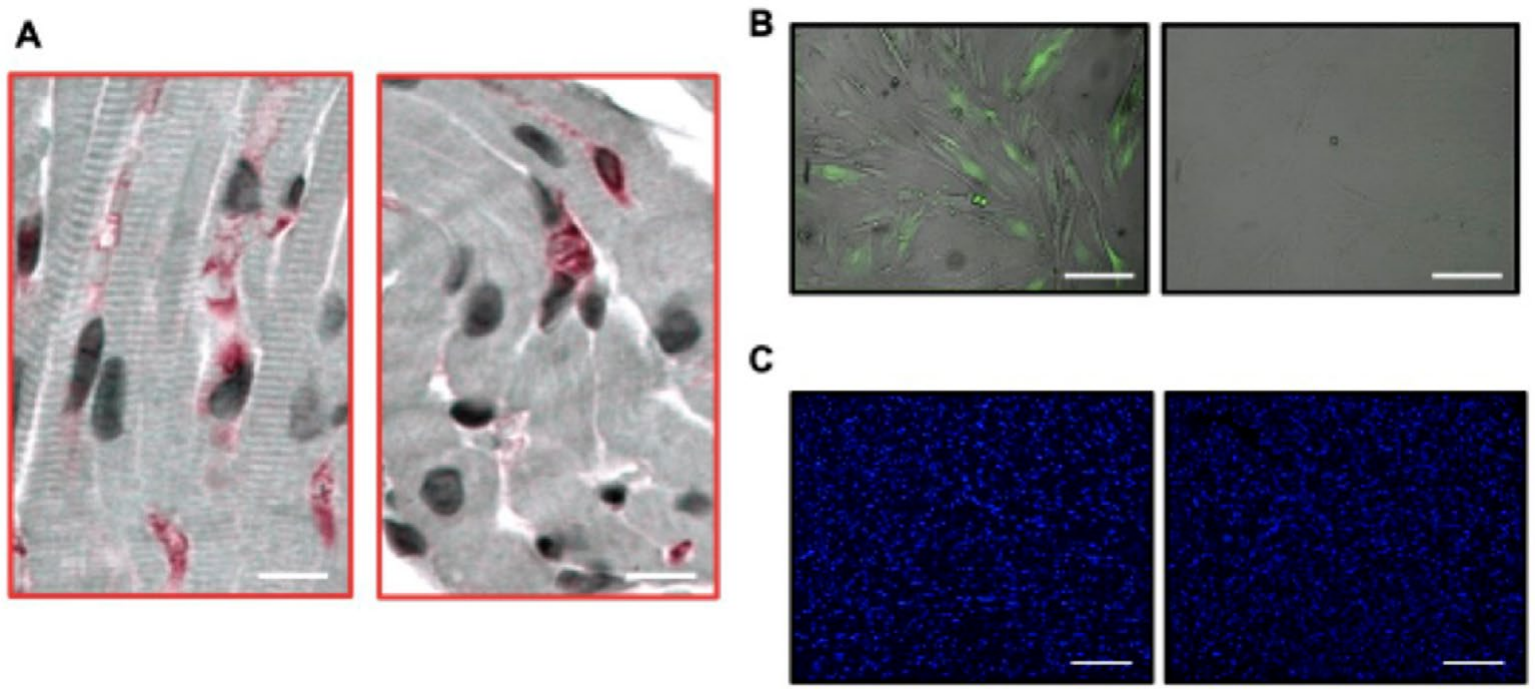
Humqn amniotic membrane–derived mesenchymal stromal cells (hAMSC) do not show successful engraftment in mouse hearts after myocardial ischemia/reperfusion (I/R). (A) Immunohistochemical detection of SSEA4, a protein expressed by hAMSC, in mouse hearts 21 days after I/R. (B) Left: Immunofluorescence detection of hAMSC transfected with a GFP‐expressing lentiviral vector (left) or an empty vector (control) (right). (C) Immunofluorescence detection of GFP‐expressing hAMSC (left) and hAMSC control (right) in mouse hearts after 21 days of I/R. Nuclei were stained with Hoechst. Scale bars 100 μm.

To determine whether hAMSC were successfully engrafted in the cardiac tissue, we analyzed the expression of the specific Mesenchymal Stem Cell protein marker SSEA4 on heart sections from the same mice as above, finding a minimal/absent homing/engraftment of these cells (Figure 3A). To exclude the possibility of hAMSC differentiation, we also generated a green fluorescent protein (GFP)‐expressing hAMSC cell line (see 2. Methods for details), thereby facilitating the detection of the signal in the hearts as well (Figure 3B). However, as with SSEA4, no GFP‐positive signal was found in the hearts injected with hAMSC (Figure 3B), indicating a minimal engraftment of hAMSC within the myocardium of mice.

### 3.3 hAMSC Expresses the Cardioprotective microRNA miR150

**FIGURE 4:**
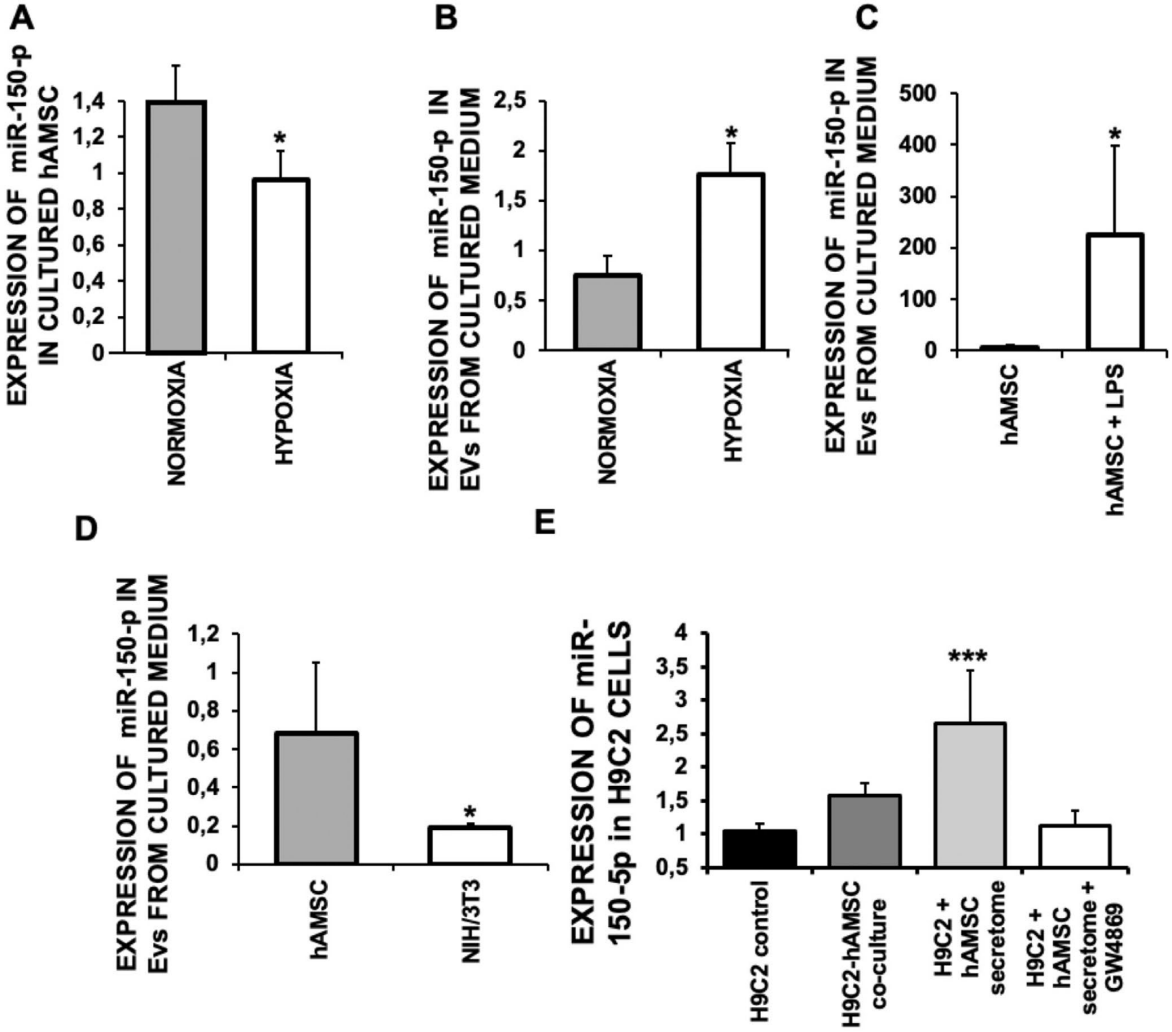
Human amniotic membrane–derived mesenchymal stromal cells (hAMSC) release extracellular vesicles (EVs) containing miR‐150‐5p under hypoxic and inflammatory culture conditions. Expression of miR‐150‐5p in cultured cells (*p* < 0.02) (A), in EVs from cultured medium under normoxic and hypoxic conditions (*p* < 0.001) (B), in EVs from cultured medium of cells treated with 500 μM (*p* < 0.03) (C), EVs from cultured medium of NIH/3 T3 cells (*p* < 0.003) (D) and H9C2 in an EVs uptake experiment (*p* < 0.001) (E). *N* = 10/group. Mean ± SD.

Our results show that hAMSC‐mediated cardioprotection does not depend on hAMSC engraftment into the myocardium, underscoring the relevance of paracrine mechanisms. Among these, microRNAs, especially miR‐150‐5p, are identified as crucial regulators of post‐infarction remodelling. Importantly, miR‐150‐5p is significantly reduced in the heart and circulating monocytes after acute myocardial infarction (AMI) in both mice and humans [10, 16, 28]. Consistent with these observations, we discovered for the first time that miR‐150‐5p levels are also decreased in cultured hAMSC under hypoxic conditions (Figure 4A).

Others found that exosomes derived from bone marrow mesenchymal stem cells (BMMSCs) can induce cardiac protection in mice subjected to myocardial infarction (26). Preclinical studies in rat models of MI have demonstrated that rat exosomes from BMMSCs, in which mR‐150‐5p was overexpressed, increased cardiac function when administered to rats after 24 h of reperfusion [29, 30]. A key finding of this work is that quantitative analysis showed a significant enrichment of miR‐150‐5p in EVs released by hAMSC under hypoxic conditions, suggesting that active packaging and secretion of miR‐150‐5p by hAMSC may be a mechanism of cardioprotection of these cells during hypoxic stress (Figure 4B). Indeed, mimicking an inflammatory response by incubating hAMSC with 500 μM bacterial LPS also elicited the release of high miR‐150‐5p‐containing EVs (Figure 4C). Notably, hAMSC‐EVs contained higher miR‐150 levels than those from other cell types, including NIH/3 T3 fibroblasts (Figure 4D). Exposure of H9C2 cardiomyoblasts to the secretome derived from hAMSC resulted in a significant increase in intracellular miR‐150‐5p levels compared with untreated controls. This elevation was consistently observed both in direct stimulation assays and in coculture conditions (H9C2:hAMSC, 1:10). In contrast, H9C2 cells treated with hAMSC secretome pre‐treated with GW4869, an inhibitor of Neutral sphingomyelinase 2 (nSMase2)‐dependent exosome biogenesis, did not exhibit any increase in miR‐150‐5p levels (Figure 4E). These findings prompted us to investigate whether EV‐mediated miR‐150‐5p delivery underlies the cardioprotective effects in vivo.

### 3.4 Cardioprotection Induced by hAMSC Is Mediated Through Regulation of the MIAT/miR‐150/HOXA‐4 Axis in Mice Subjected to I/R

**FIGURE 5:**
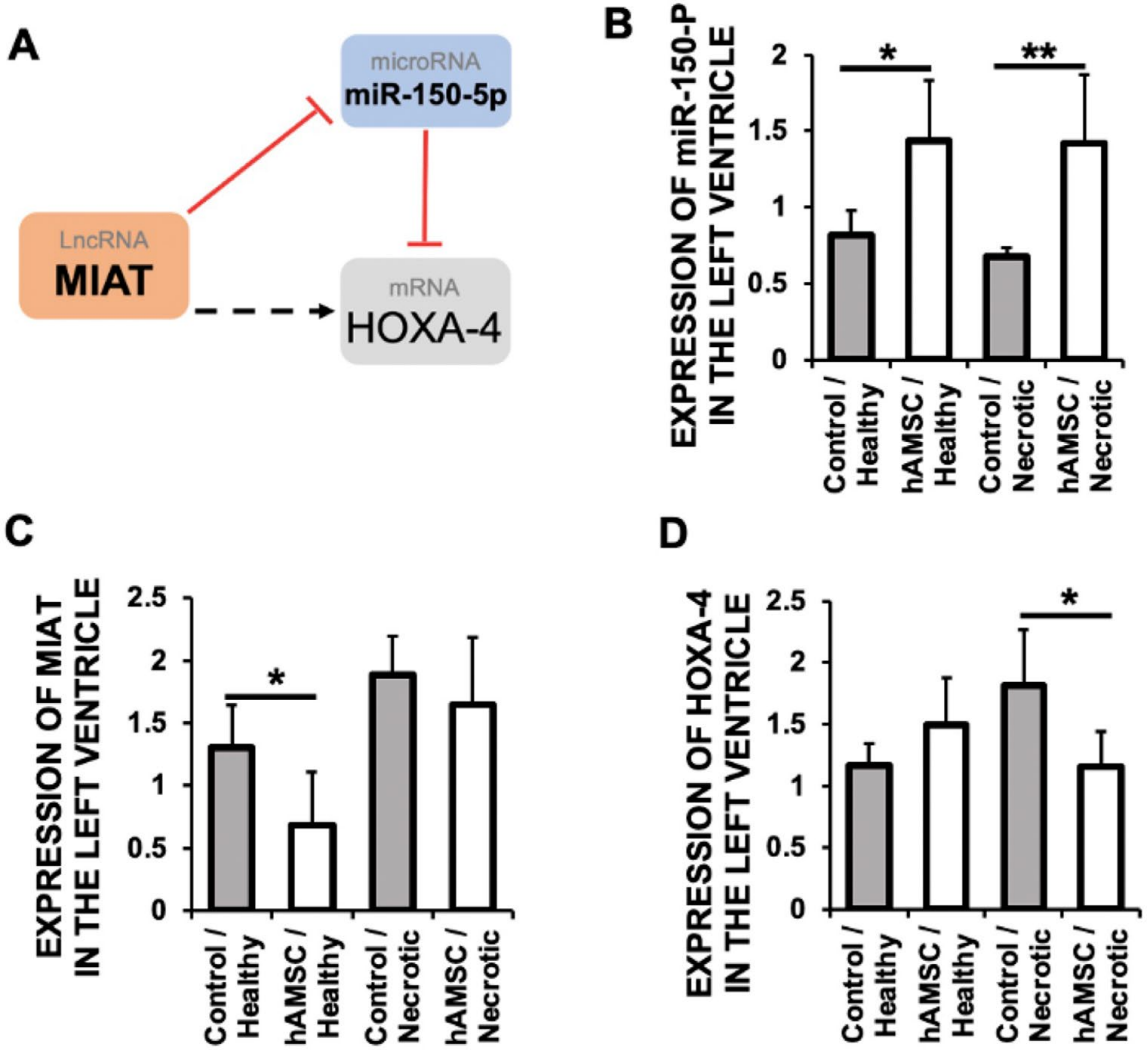
Human amniotic membrane–derived mesenchymal stromal cells (hAMSC) regulate the axis MIAT/miR‐150‐5p/HOXA‐4 in the hearts of mice subjected to myocardial ischemia/reperfusion (I/R). (A) Schematic representation of the MIAT/miR‐150‐5p/HOXA‐4 axis. (B–D) Expression of miR‐150‐5p (*p = 0.005; **p = 0.003) (B), MIAT (*p = 0.0293) (C), and HOXA‐4 (*p = 0.0338) (D) in the healthy and necrotic areas of the left ventricle of mice under I/R (control) and injected with 2 × 10^5^ hAMSC. *N* = 10 mice/group. Mean ± SD.

Long non‐coding RNAs (lncRNAs), such as MIAT (myocardial infarction–associated transcript), have been implicated in post‐infarction remodelling and may act as upstream regulators of miRNA activity [12, 14, 27]. In this context, MIAT has been reported to function as a competitive endogenous RNA (ceRNA), modulating the bioavailability of miR‐150, which targets HOXA‐4 [16], a profibrotic transcription factor in post‐MI adverse remodelling (Figure 5A).

Two days after reperfusion, injection of hAMSC significantly increased miR‐150 levels in cardiac tissue, including healthy and necrotic myocardial regions (Figure 5B). Concurrently, MIAT expression was markedly reduced in the hearts of treated mice (Figure 5C). These findings suggest that hAMSC may attenuate adverse cardiac remodelling by downregulating MIAT, thereby enhancing miR‐150 availability, at least in the healthy area of heats subjected to I/R. This regulatory shift may underlie the significant reduction in myocardial fibrosis observed in hAMSC‐treated animals (Figure 2B), which is consistent with the downregulation of HOXA‐4 in these mice (Figure 5D).

**FIGURE 6:**
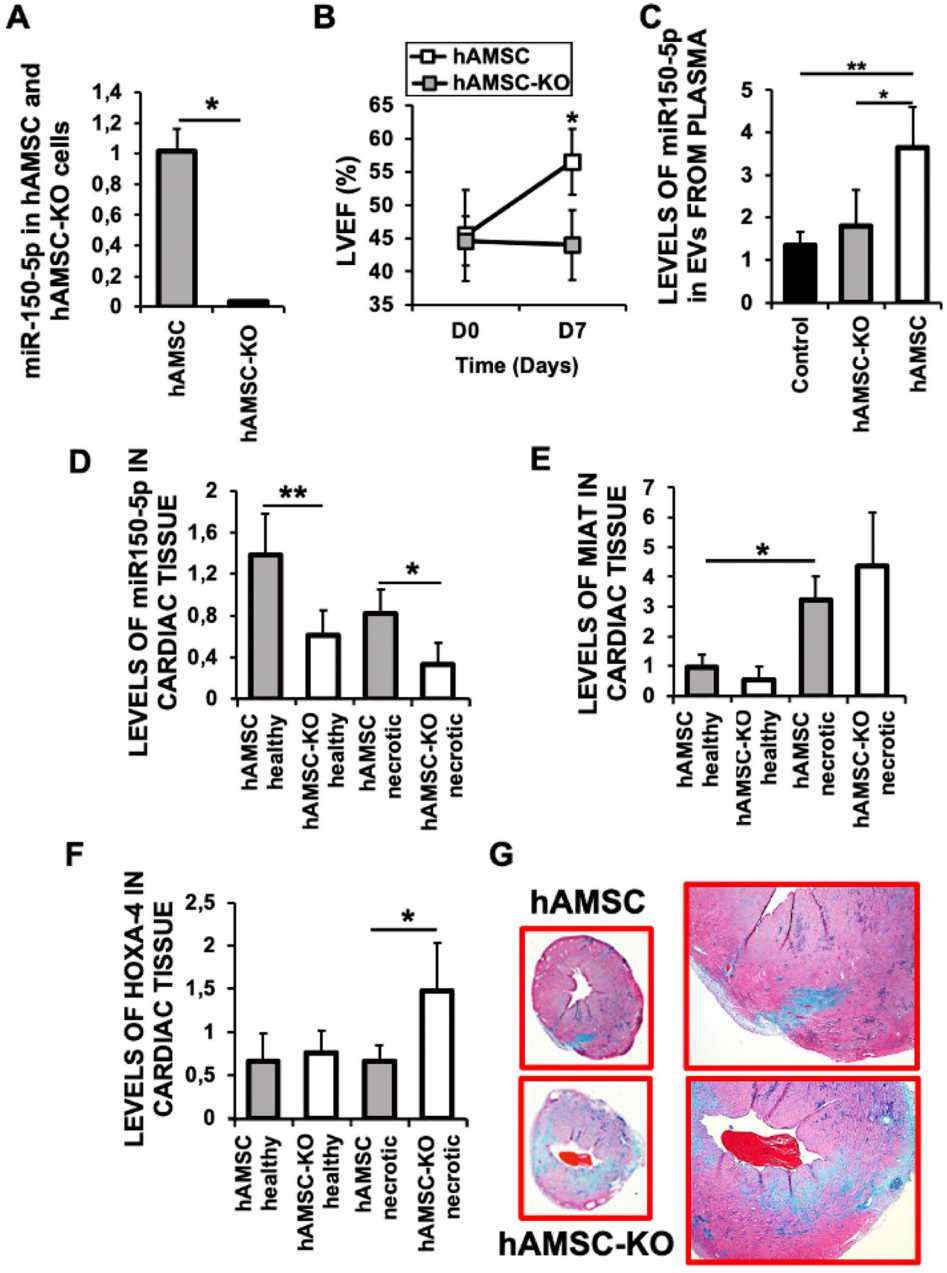
Mice treated with human amniotic membrane–derived mesenchymal stromal cells lacking miR‐150‐5p (hAMSC‐KO) shows reduced Left ventricular ejection fraction (LVEF) after myocardial ischemia/reperfusion (I/R), expresses less cardiac miR‐150‐5p, along with increased fibrotic HOXA‐4 levels, and extensive cardiac fibrosis by Day 21 after reperfusion. (A) Expression of miR‐150‐5p in cultured hAMSC and hAMSC‐KO (*p* < 0.0001). (B) LVEF of hearts of mice after 7 days of I/R, and previously injected with 2 × 10^5^ hAMSC or hAMSC‐KO by day 2 after reperfusion (*p < 0.002 D7). (C) Levels of miR‐150‐5p after 21 days of I/R in extracellular vesicles (EVs) isolated from plasma of mice injected with hAMSC or hAMSC‐KO by day 2 after I/R (*p < 0.03 hAMSC vs. Control and hAMSC vs. hAMSC‐KO). (D–F) Cardiac levels of miR‐150‐5p (*p < 0.012, **p < 0.001) (D), MIAT (*p < 0.02) (E), and HOXA‐4 (*p < 0.001) (F) in the hearts of mice subjected to I/R and injected with 2 × 10^5^ hAMSC, and hAMSC‐KO. (G) Masson's Trichromic staining of heart sections isolated by Day 21 after I/R and injected after 2 days of reperfusion with hAMSC, or hAMSC‐KO. *N* = 10 mice/group. Mean ± SD.

To test whether miR‐150‐5p is indeed a mechanism elicited by hAMSC in cardiac protection, we used CRISPR/Cas9 genome editing to silence microRNA miR‐150‐5p in hAMSC (hAMSC‐KO) (Figure 6A). Mice subjected to I/R and injected with hAMSC‐KO exhibited a notable reduction of cardiac function, as evidenced by a reduced LVEF at Day 7 post‐reperfusion, compared to those receiving miR‐150‐5p expressing hAMSC (Figure 6B). Together, these results confirm and further support a critical role for miR‐150‐5p as a molecular effector underlying the cardioprotective effects of hAMSC‐EVs after myocardial infarction. Indeed, circulating blood levels of miR‐150‐5p containing EVs from mice injected with hAMSC‐KO were markedly decreased when compared to mice injected with hAMSC containing miR‐150 (Figure 6C). In addition, cardiac tissue from mice treated with hAMSC‐KO exhibited significantly reduced levels of miR‐150 (Figure 6D), in contrast to the elevated levels observed following injection of wild‐type hAMSC (Figure 5B). Consistent with these findings, increased HOXA4 levels (Figure 6E) were associated with greater fibrosis in mice injected with hAMSC‐KO (Figure 6F), further supporting the role of miR‐150‐5p as a key regulator of anti‐fibrotic and cardioprotective responses after ischemic injury.

To functionally validate hAMSC‐induced HOXA4 expression, and given the profibrotic role of HOXA4 and its transcriptional control of GSK3β, we used GSK3β as a readout of the HOXA4–GSK3β/Wnt axis and quantified its expression in post‐I/R infarct tissue versus healthy myocardium. In vivo, infarcted hearts from hAMSC‐injected animals showed a significant reduction in GSK3β levels, confirming functional downregulation of HOXA4 in this context (Figure S2).

### 3.5 EVs Derived From hAMSC Confer Cardioprotection in Mice Following I/R

**FIGURE 7:**
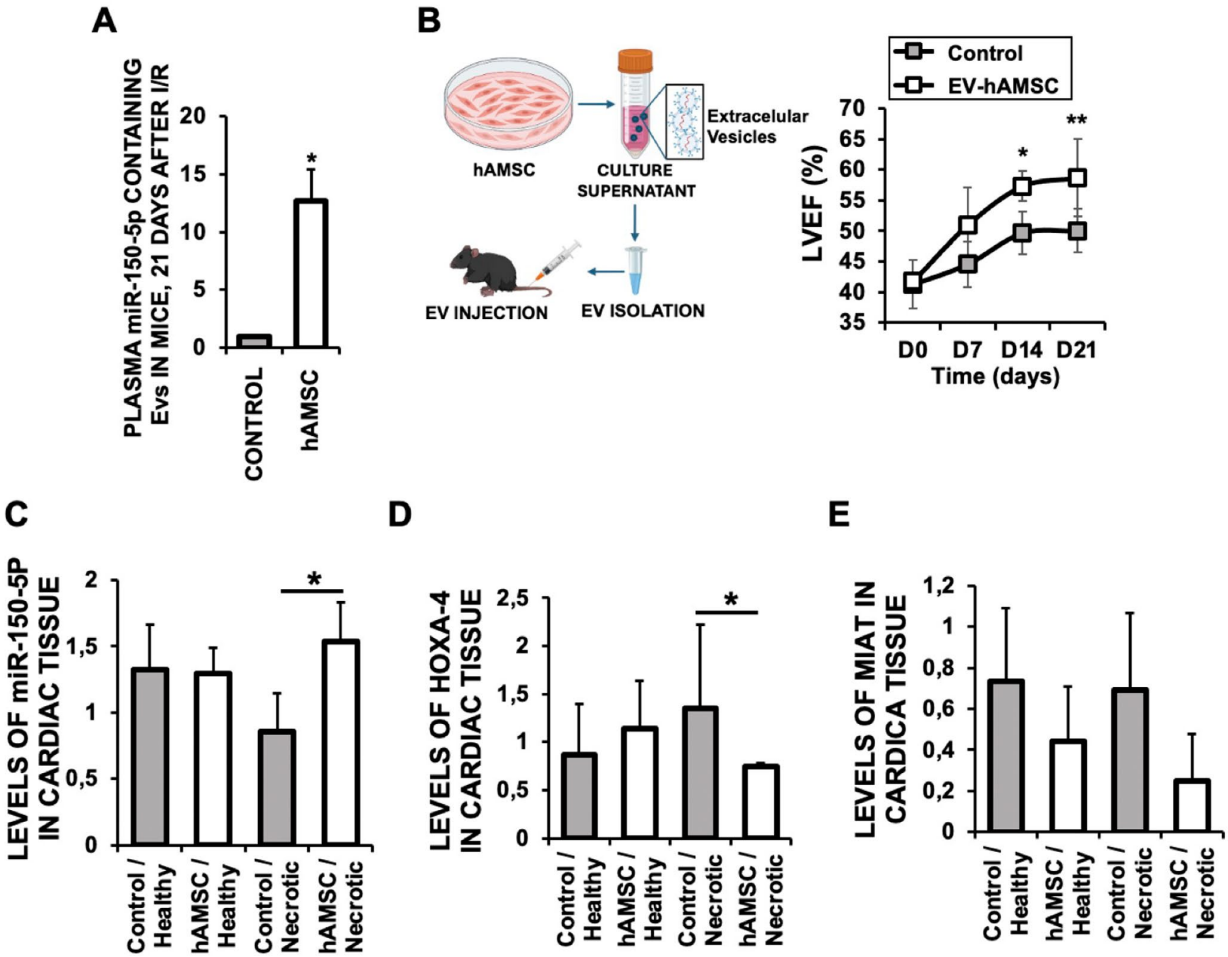
Injection of miR‐150‐5p‐containing extracellular vesicles (EVs) induces cardiac protection in mice subjected to myocardial ischemia/reperfusion (I/R). (A) miR‐150‐5p in EVs from mouse plasma after 21 days of I/R, injected with 2 × 10^5^ human amniotic membrane–derived mesenchymal stromal cells (hAMSC) by Day 2 after reperfusion (*p* < 0.0001). (B) Left. Schematic representation. Right. Left ventricular ejection fraction (LVEF) of mouse hearts subjected to I/R, and injected with EVs containing miR‐150‐5p by day 2 after reperfusion, at the times indicated. (*p < 0.033, and **p < 0.062 Control vs. EV‐hAMSC respectively). (C–E) Expression of miR‐150‐5p (*p < 0.027 Necrotic Control vs. Necrotic EVs from hAMSC) (C), HOXA‐4 (*p < 0.01 Necrotic Control vs. Necrotic EVs from hAMSC) (D), and MIAT (E) in the healthy and necrotic areas of the hearts of mice after 21 days of I/R, and injected with EVs‐containing miR‐150‐5p by Day 2 after reperfusion. *N* = 10 mice/group. Mean ± SD.

Our data show that hAMSC reduce the levels of HOXA‐4 in the hearts of mice subjected to I/R, and it correlates with the increased expression of miR‐150 (Figure 5). Indeed, extensive blood circulation of miR‐150‐5p‐containing EVs was found in mice subjected to I/R by Day 21 after injection of hAMSC (Figure 7A). To determine whether the beneficial effects of hAMSC on adverse cardiac remodelling are mediated through the delivery of miR‐150‐5p‐containing EVs (Figure 4B), we administered miR‐150‐enriched EVs, rather than hAMSC, into mice subjected to ischemia/reperfusion (I/R) injury. This treatment resulted in a significant and sustained improvement in cardiac function over time (Figure 7B), accompanied by increased levels of miR‐150‐5p in the necrotic regions of the hearts (Figure 7C). Consequently, HOXA‐4 levels, which are negatively regulated by miR‐150, were significantly decreased (Figure 7D), while MIAT expression remained unchanged (Figure 7E), indicating a direct effect of miR‐150‐5p delivered by the injected EVs in mice.

Collectively, our findings provide mechanistic evidence supporting a causal role for EV‐miR150‐5p derived from human amniotic membrane mesenchymal stem cells, hAMSC, beyond 24 h of reperfusion to prevent adverse cardiac remodelling, and thereby underscoring their potential as a novel exosome‐based strategy for human cardiac repair through MIAT/HOXA4 modulation.

## 4 Discussion

This study presents mechanistic evidence of the cardioprotective effects of hAMSCs in a mouse model of myocardial ischemia/reperfusion (I/R). hAMSC treatment improves cardiac function, reduces inflammatory cell infiltration, and attenuates myocardial fibrosis. Importantly, we identify the MIAT/miR‐150‐5p/HOXA4 regulatory axis as a key mediator of these effects. To further explore the role of miR‐150‐5p, we administered CRISPR–Cas9–edited hAMSCs lacking miR‐150‐5p (hAMSC‐KO); in these animals, the cardioprotective benefits of hAMSCs were abolished, underscoring the pivotal contribution of miR‐150‐5p to therapeutic efficacy. Moreover, miR‐150‐5p–containing EVs isolated from hAMSCs (hAMSC‐EVs) conferred significant cardioprotection 7 days after myocardial I/R. Together, these findings substantiate the therapeutic potential of hAMSCs in myocardial infarction and highlight the MIAT/miR‐150‐5p/HOXA4 axis, and hAMSC‐derived EVs targeting this pathway, as a promising basis for advanced, cell‐free cardioprotective therapies.

Prior studies have reported cardioprotective or cardioactive effects of extracellular vesicles/secretome derived from human amniotic fluid‐derived stromal/stem cells in rodent ischemic settings, including protection in a rat I/R model when small EVs are administered prior to reperfusion [28] and transient stimulation of cardiomyocyte cell‐cycle activity in a neonatal mouse myocardial infarction model [10]. Our findings extend this body of work by evaluating an amniotic membrane–derived stromal cell product in an adult I/R context and by providing mechanistic insight implicating the MIAT/miR‐150‐5p/HOXA4 regulatory axis.

Our study highlights the critical importance of timing in the administration of hAMSC following I/R. Mice treated within 2 days of I/R, corresponding to the transition from acute inflammation to the onset of tissue remodelling, exhibited enhanced functional recovery compared with those treated at later time points. Our data highlight the early post‐infarction phase, marked by cell death, inflammation, and ECM remodelling, as a clinically therapeutic window in which hAMSC demonstrate peak reparative potential [27]. In contrast, administering at later stages, when fibrotic and scar tissue predominate, markedly diminishes their potential [17].

Mesenchymal stem cells (MSCs) derived from adult tissues, such as bone marrow and adipose tissue, have shown promising effects in enhancing myocardial recovery following ischemia/reperfusion (I/R) injury in preclinical models [7, 20, 31]. However, despite these encouraging results, their clinical translation remains limited due to challenges such as invasive harvesting procedures, donor variability, and potential immunogenicity. hAMSC sythesize a broad array of factors involved in angiogenesis, including VEGF, HGF, PDGF, and TGF‐β, tissue remodelling MMPs, matrix regulatory TIMPs, and immune modulation modulatory IL‐6, IL‐11, LIF proteins [3, 7], conferring cardioprotective effect. Their immunomodulatory profile, non‐invasive sourcing, and high proliferative capacity further enhance their appeal as a clinically therapeutic option.

Prior work has implicated EV‐associated miR‐150‐5p as a cardioprotective signal in preclinical I/R settings, including studies in rats using bone marrow–derived MSC EVs enriched for miR‐150‐5p and reporting attenuation of remodelling alongside changes in downstream pathways such as TXNIP [20]. In this context, our results are not mutually exclusive and extend the field in three important ways. First, we demonstrate efficacy using extracellular vesicles derived from human amniotic membrane MSCs in a mouse I/R model, supporting the translational relevance of this understudied cell source. Second, rather than relying on miRNA enrichment/overexpression approaches, we provide genetic loss‐of‐function evidence for necessity: CRISPR/Cas9‐mediated deletion of miR‐150‐5p in hAMSCs generates miR‐150‐5p–deficient EVs that fail to reproduce the functional and anti‐fibrotic benefits observed with wild‐type EVs, our data delineate a mechanistic regulatory axis linking EV‐miR‐150‐5p to the long non‐coding RNA MIAT and the transcription factor HOXA4, thereby refining the molecular framework through which EV‐miR‐150‐5p contributes to cardioprotection.

Two additional features position hAMSC as therapeutic effectors in the clinical setting: hAMSC express low levels of MHC class I molecules [32, 33, 34] and lack the co‐stimulatory molecules necessary for T cell activation [35]. This immune‐evasive phenotype minimizes recognition and rejection by the host immune system, enabling hAMSC to deliver signals to the injured myocardium without eliciting a strong immune response [32]. Such immune privilege positions hAMSC as a particularly attractive ‘off‐the‐shelf’ therapeutic option, thereby preventing the need for patient‐specific cell preparation or immunosuppressive therapy.

hAMSC have also demonstrated the ability to express early and late cardiomyogenic markers (GATA4, cardiac troponin T, ANP), particularly when exposed to cardiogenic growth factors or co‐cultured with cardiac cells [36, 37, 38]. In vivo transplantation studies have similarly reported hAMSC expressing cardiomyocyte‐specific proteins within infarcted myocardium, suggesting a limited capacity for in situ trans‐differentiation [37]. This is further supported by studies showing that hAMSC‐derived exosomes carry cardioprotective and lineage‐associated factors [35]. Together, these findings indicate that hAMSC retain the ability to adopt cardiomyogenic phenotypes under specific conditions, although their full differentiation into functional cardiomyocytes remains a subject of ongoing debate. Our findings are consistent with the consensus that hAMSC do not differentiate into fully contractile cardiomyocytes in vivo [31]. Instead, their therapeutic benefits appear to be primarily mediated through paracrine mechanisms. This is further supported by numerous studies demonstrating the ability of MSCs to reduce fibrosis and promote tissue repair in liver, lung, bone and skin diseases [32, 33, 34, 39, 40].

A previously undescribed pathological fibrotic pathway in the myocardium has been characterized, revealing that elevated levels of MIAT, a long non‐coding RNA, are associated with increased susceptibility to heart failure [16], while at the same time, microRNA miR‐150‐5p plays a protective role by preventing cardiac fibrosis and apoptosis, key processes in maladaptive myocardial remodelling after infarction [28]. miR‐150‐5p is reduced in failing hearts, partly due to sequestration by MIAT, and identified Homeobox A4 (HOXA‐4), a transcription factor involved in embryonic development and fibrosis, as a direct target of the MIAT/miR‐150‐5p interaction. Specifically, MIAT overexpression increases HOXA‐4 levels, whereas miR‐150‐5p overexpression directly represses HOXA‐4, mitigating its pro‐fibrotic effects. Here, for the first time we further unveil that the MIAT/miR‐150‐5p/HOXA‐4 axis mediates the cardioprotective effects of hAMSC‐EVs in a murine model of I/R.

Exosomes have played a central role in mediating cardiac repair in murine models of MI [9, 10, 41, 42]. These nanosized vesicles encapsulate and deliver bioactive molecules, including microRNAs, proteins, and lipids, to recipient cells in the injured myocardium, modulating key reparative pathways [18]. Here we show that hAMSC secrete EVs carrying miR‐150‐5p in vitro in conditions simulating ischemic heart, such as hypoxia and inflammatory conditions, and in vivo, which strongly implicates this microRNA as a pivotal mediator of its therapeutic effects. Notably, targeted deletion of miR‐150‐5p in hAMSCs using CRISPR‐Cas9 technology (hAMSC‐KO) significantly attenuated their cardioprotective effects, underscoring a major role of this microRNA in orchestrating the reparative response. These findings not only establish a causal link between miR‐150‐5p expression and therapeutic efficacy but also highlight its potential as a molecular target to enhance cell‐based therapies for myocardial injury.

miR‐150‐5p contributes to the immunomodulatory properties of hAMSC exosomes, as evidenced by its ability to inhibit STAT1 and NF‐κB signalling pathways, reducing the production of pro‐inflammatory cytokines such as TNF‐α and IL‐6 in response to inflammatory stimuli [35]. These effects highlight miR‐150‐5p as a potent therapeutic molecule delivered via hAMSC‐derived exosomes [35, 43]. Based on genome‐wide association studies (GWAS) and prior transcriptomic analyses implicating the MIAT/miR‐150‐5p/HOXA‐4 axis in myocardial injury, we specifically focused on miR‐150‐5p as a candidate mediator of hAMSC reparative effects. However, it is essential to note that hAMSC secrete a broad range of bioactive molecules, including additional microRNAs, cytokines, growth factors, and extracellular vesicles, that likely contribute to their regenerative potential beyond miR‐150‐5p alone [17, 31, 44]. Indeed, the benefits of hAMSC are not limited to myocardial infarction, since they have shown a positive effect in other cardiovascular conditions, such as ischemic limb disease and pulmonary hypertension, where their paracrine actions promote angiogenesis, attenuate fibrosis, and modulate inflammation [1, 44], and in the nervous system, where they promote axon growth and restore neuronal activity, further underscoring their versatility as a regenerative therapy, while showing possible implications in electrical cell activities [1].

To the best of our knowledge, this is the first in vivo study to demonstrate that hAMSC‐derived exosomes serve as vehicles for miR‐150‐5p delivery to modulate this pathological axis. This positions miR‐150‐5p not only as a critical regulator of post‐injury remodelling but also as a promising target for developing cell‐free, miRNA‐based therapeutic strategies aimed at enhancing cardiac regeneration. However, while this study represents a novel significant step forward, additional research is needed to identify new EV cargo components and to investigate the long‐term effects of hAMSC therapy. Such studies will be essential for validating these findings and paving the way for future clinical use of cell‐free therapeutic strategies.

## Author Contributions

**Nunzio Alcharani:** research, data collection, data analysis. **Laura Tesoro:** research. **Javier Díez‐Mata:** research. **José Luis Zamorano:** interpretation of data, revising for intellectual content. **Marta Saura:** data analysis, interpretation of data. **Maite Iglesias:** work design, revision, final approval of the version to be published. **Carlos Zaragoza:** conception of the work, research, data collection, data analysis, drafting the work, revising the work, final approval of the version to be published, and agreement to be accountable for all aspects of the work.

## Funding

The present work was funded by the Universidad Francisco de Vitoria Grant (UFV 2023).

## Ethics Statement

All animal procedures were performed upon approval from the institutional and governmental agencies as stated in the 2. Materials and Methods Section.

## Conflicts of Interest

The authors declare no conflicts of interest.

## Supporting information


**Figure S1:** Validation and analysis of isolated Exosomes using the Nanosight LM10 system. (A) Western blot analysis of Calnexin, HSP70, CD63, TSG101, CD81 and CD9 in exosomes derived from hAMSC. (B) Images obtained from H_2_O and PBS as negative controls, 100 nm nanoparticles as positive controls, and exosome analysis derived from human amniotic membrane–derived mesenchymal stromal cells (hAMSC), NIH/3 T3 cells, and mouse plasma. (C) Automated quantification of extracted signals from the system, expressed as the average Finite Track Length Adjustment (FTLA) in terms of concentration/size.

**Figure S2:** Expression of GSK3β in response to administration of human amniotic membrane–derived mesenchymal stromal cells (hAMSC) in mice subjected to myocardial ischemia/reperfusion (I/R). (A) Schematic representation of the MIAT‐miR‐150‐5p‐HOXA‐4 and the readout GSK3β. (B) Expression of GSK3β in mouse hearts injected with hAMSC (see Methods for details) and subjected to I/R. *N* = 10 mice/group. Mean ± SD. **p < 0.002 Control vs. hAMSC subjected to I/R.

## Data Availability

The data that support the findings of this study are available from the corresponding author upon reasonable request.
