# Supplementary figures and images for: Identification of miR‐150‐5p in Human Amniotic Membrane Mesenchymal Cell‐Derived Extracellular Vesicles as a Novel Mechanism Driving Cardioprotection
Source: Eur J Clin Invest. 2026 Apr 28;56:e70212. doi: 10.1111/eci.70212 (PMC13123203; doi:10.1111/eci.70212)

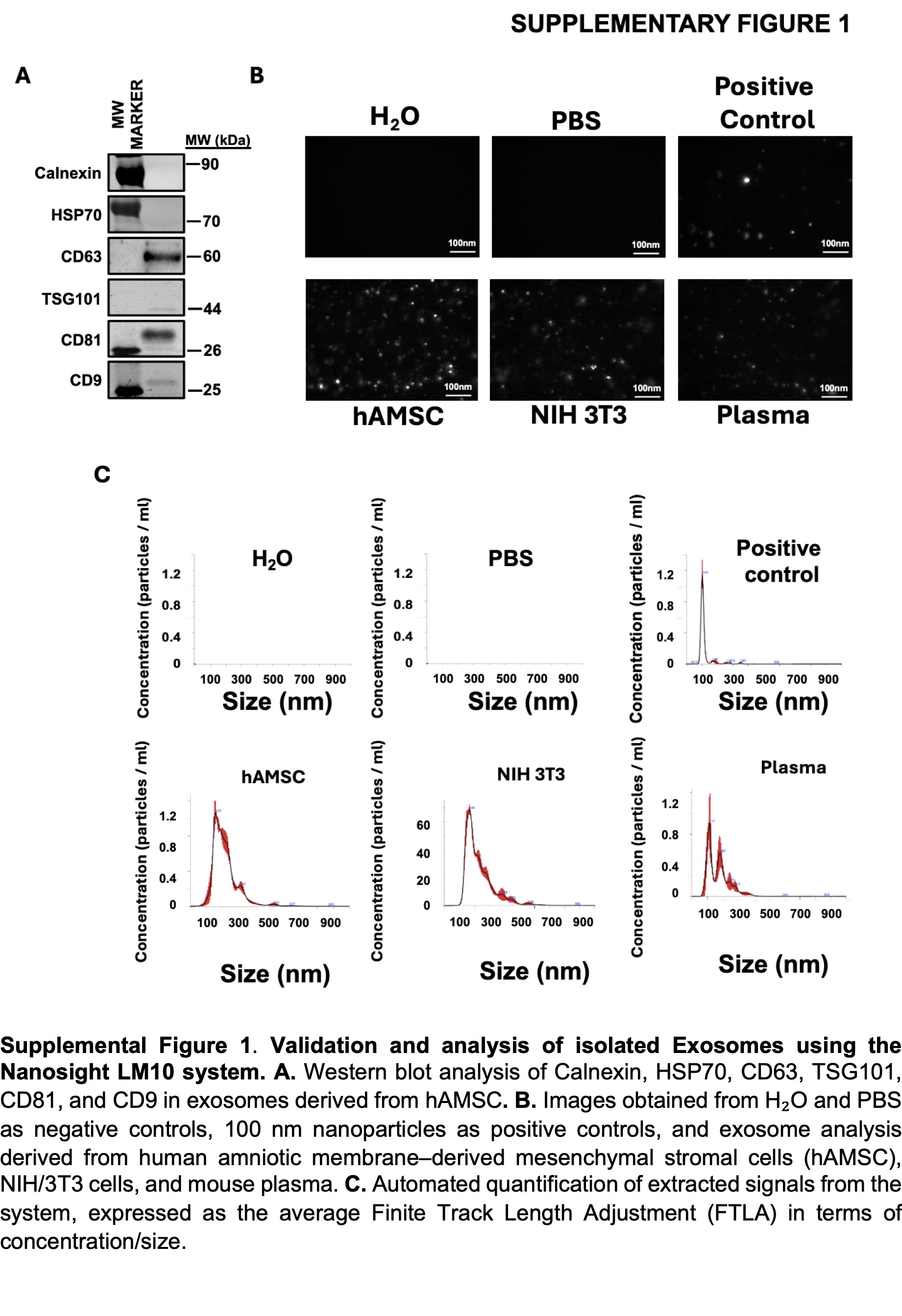

Supplement: Supplementary file 1 — Figure S1: Validation and analysis of isolated Exosomes using the Nanosight LM10 system. (A) Western blot analysis of Calnexin, HSP70, CD63, TSG101, CD81 and CD9 in exosomes derived from hAMSC. (B) Images obtained from H2O and PBS as negative controls, 100 nm nanoparticles as positive controls, and exosome analysis derived from human amniotic membrane–derived mesenchymal stromal cells (hAMSC), NIH/3 T3 cells, and mouse plasma. (C) Automated quantification of extracted signals from the system, expressed as the average Finite Track Length Adjustment (FTLA) in terms of concentration/size. [file ECI-56-e70212-s002.jpg]

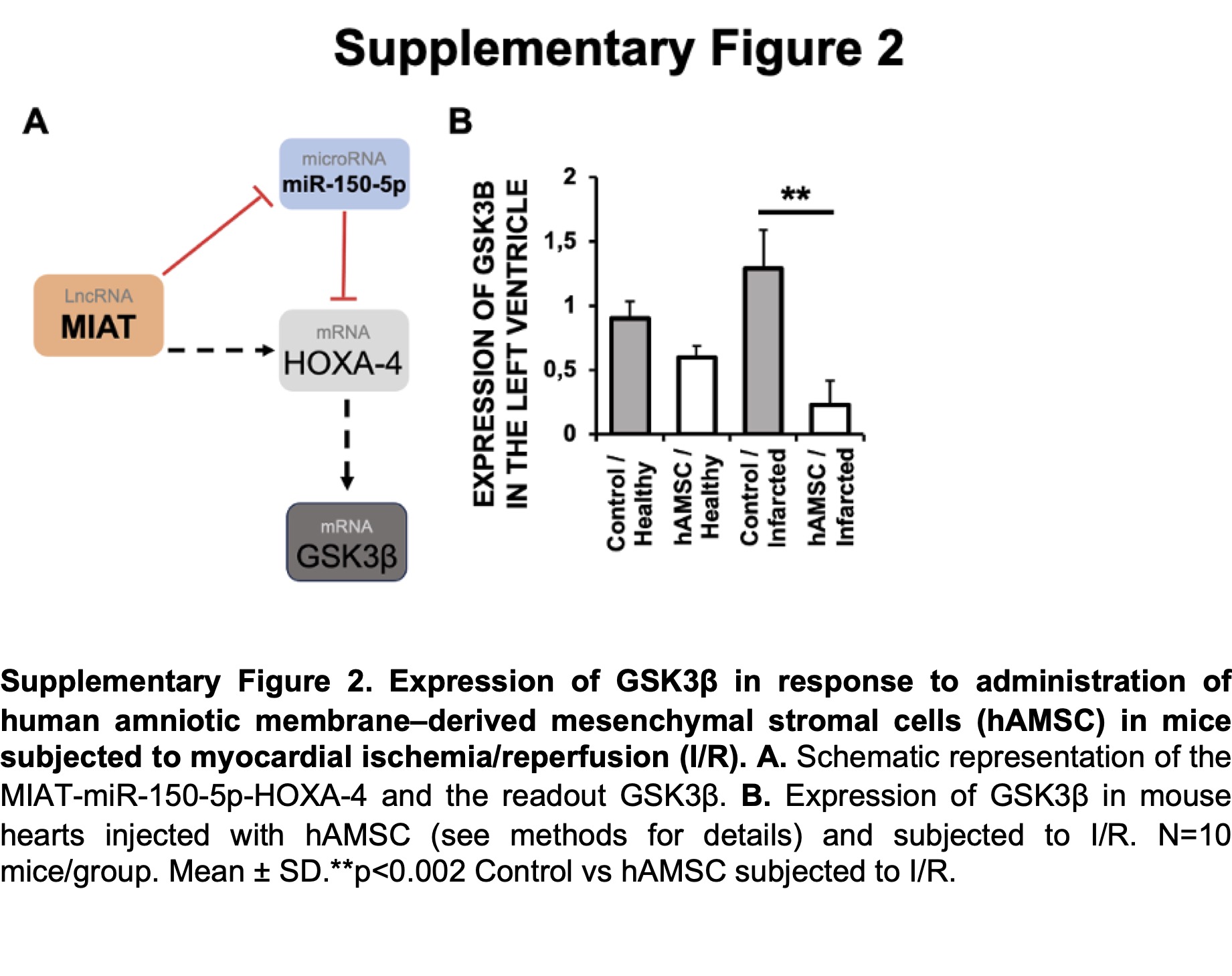

Supplement: Supplementary file 2 — Figure S2: Expression of GSK3β in response to administration of human amniotic membrane–derived mesenchymal stromal cells (hAMSC) in mice subjected to myocardial ischemia/reperfusion (I/R). (A) Schematic representation of the MIAT‐miR‐150‐5p‐HOXA‐4 and the readout GSK3β. (B) Expression of GSK3β in mouse hearts injected with hAMSC (see Methods for details) and subjected to I/R. N = 10 mice/group. Mean ± SD. **p < 0.002 Control vs. hAMSC subjected to I/R. [file ECI-56-e70212-s001.jpg]
